# Calpain: the regulatory point of myocardial ischemia-reperfusion injury

**DOI:** 10.3389/fcvm.2023.1194402

**Published:** 2023-06-29

**Authors:** Guo-Yang Liu, Wan-Li Xie, Yan-Ting Wang, Lu Chen, Zhen-Zhen Xu, Yong Lv, Qing-Ping Wu

**Affiliations:** ^1^ Department of Anesthesiology, Union Hospital, Tongji Medical College, Huazhong University of Science and Technology, Wuhan, China; ^2^ Institute of Anesthesia and Critical Care Medicine, Union Hospital, Tongji Medical College, Huazhong University of Science and Technology, Wuhan, China; ^3^ Key Laboratory of Anesthesiology and Resuscitation (Huazhong University of Science and Technology), Ministry of Education, Wuhan, China

**Keywords:** programmed cell death, myocardial ischemia-reperfusion injury, calpain, apoptosis, parthanatos

## Abstract

Calpain is a conserved cysteine protease readily expressed in several mammalian tissues, which is usually activated by Ca^2+^ and with maximum activity at neutral pH. The activity of calpain is tightly regulated because its aberrant activation will nonspecifically cleave various proteins in cells. Abnormally elevation of Ca^2+^ promotes the abnormal activation of calpain during myocardial ischemia-reperfusion, resulting in myocardial injury and cardiac dysfunction. In this paper, we mainly reviewed the effects of calpain in various programmed cell death (such as apoptosis, mitochondrial-mediated necrosis, autophagy-dependent cell death, and parthanatos) in myocardial ischemia-reperfusion. In addition, we also discussed the abnormal activation of calpain during myocardial ischemia-reperfusion, the effect of calpain on myocardial repair, and the possible future research directions of calpain.

## 1. Introduction

Heart disease is the leading cause of death, and coronary heart disease (CHD) accounts for a large proportion of deaths (1). Mammalian terminally differentiated cardiomyocytes have limited self-replacement capacity and cannot be replenished after injury, resulting in cardiac dysfunction and heart failure (2). Coronary artery recanalization and coronary artery bypass grafting provide the most effective treatment for patients with cardiac artery occlusion (3), reduced heart damage during ischemia. However, the ischemic heart will have a series of pathophysiological reactions after blood supply reconstruction, which will worsen myocardial damage, cause cardiomyocyte death, and is known as reperfusion injury. Cardiac reperfusion injury has not been effectively solved. How to reduce reperfusion injury and reduce the occurrence of heart failure caused by reperfusion injury is an urgent clinical problem to be solved. Calcium overload during myocardial ischemia-reperfusion (I/R) is one of the crucial mechanisms of myocardial reperfusion injury (4). Calcium overload can activate calcium-dependent protein hydrolases, such as calpain, a cysteine protease, which can nonspecifically cleave various proteins, leading to cell damage and death. Cell death is the central link of ischemia-reperfusion injury. Cell death can be subdivided into programmed cell death (PCD) and non-programmed cell death. Unlike non-programmed cell death, such as classical necrosis, PCD depends on specific molecular mechanisms (5). This provides the foundation for interventions to reduce injury. Accumulating evidence indicates that calpain regulates PCD and is critically associated with myocardial I/R injury (6). Therefore, we summarize the role of calpain in various PCD in myocardial I/R injury and stress that the pathways of PCD are complex and interact under the action of calpain. In addition, we also described the process of abnormal activation of calpain caused by the increase of intracellular Ca^2+^ during I/R, the effect of calpain on myocardial repair, analyzed the possible reasons for different results of inhibiting calpain in the basic experiment, and proposed the possible future research directions of calpain.

## 2. Abnormal activation of calpain during myocardial ischemia-reperfusion

### 2.1. Abnormal elevation of calcium concentration in ischemic reperfusion myocardium

Ca^2+^ acts as the second messenger and participates in multiple biological processes in cells. Ca^2+^ plays a decisive role in the systolic function of the heart. Generally, the intracellular Ca^2+^ content is much lower than that in extracellular (7). In normal physiological conditions, intracellular Ca^2+^ content is usually lower than 0.05 μmol/L (8). Ca^2+^ in cardiomyocytes mainly comes from extracellular and sarcoplasmic reticulum (SR) (9). When the myocardial cell membrane is depolarization, the L-type Ca^2+^ channels (LTCC) on the cell membrane are open, issues in the flow of extracellular Ca^2+^ into the cells. When intracellular Ca^2+^ concentration is higher than 10 μmol/L, ryanodine receptors (RyR) in the SR are activated, Ca^2+^ in SR flow to the cytoplasm, which is called Ca^2+^-induced Ca^2+^ release (10). Ca^2+^-induced Ca^2+^ release is the core of the calcium signal transduction mechanism (11). In general, the increase of Ca^2+^ will lead to myocardial contraction, and then Ca^2+^ in the myocardial diastolic phase is transported back to the SR through SR Ca^2+^ ATPase (SERCA) or transferer to extracellular through Na^+^/Ca^2+^ exchanger (NCX) in the plasma membrane. However, during ischemia, ATP deficiency inhibited Na^+^/K^+^-ATPase and reduced Na^+^ excretion, anaerobic glycolysis led to cytoplasmic acidification, and the increased H^+^ was discharged through Na^+^/H^+^ exchanger (NHX) to promote extracellular Na^+^ influx. Together with Na^+^/HCO_3_^−^ cotransporter and persistent Na^+^ channels, all mechanisms increase intracellular Na^+^ content (12). Abnormally elevated intracellular Na^+^ changed the membrane potential leading to NCX reverse exchange, which increased intracellular Ca^2+^ concentration (13). Moreover, the reperfusion blood flow took away the extracellular H^+^, which led to increased gradient difference of H^+^ between intracellular and extracellular. The combined effects were strengthened, and the intracellular Ca^2+^ concentration would further increase (6). In addition, studies have shown that Ca^2+^-sensitive RyR is disrupted during myocardial I/R (14). The leakage of Ca^2+^ in the SR is also the cause of calcium overload.

### 2.2. Elevated calcium hyperactivate calpains

**Figure 1:**
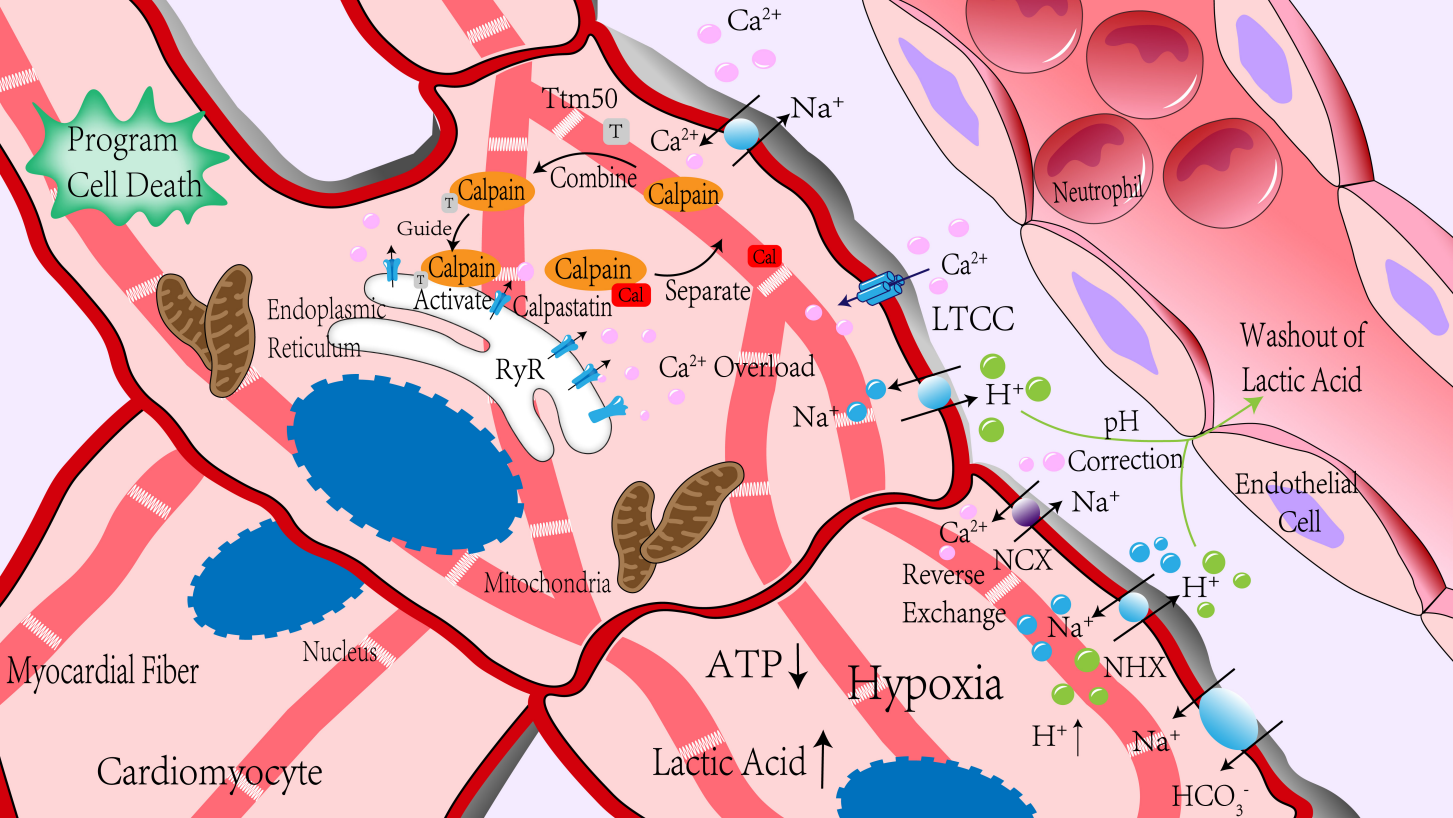
Calpain was significantly activated during ischemia-reperfusion. In the process of myocardial ischemia, due to anaerobic metabolism, ATP production is insufficient, lactic acid production is increased, pH value is decreased, H^+^ content is increased, Na^+^/H^+^ exchange is increased, and ATP deficiency leads to the insufficient activity of channel protein that mediates Na^+^ outflow, so intracellular Na^+^ content is increased. Changes in membrane potential caused by changes in Na^+^ content led to reverse exchange of Na^+^/Ca^2+^ exchanger, and ischemia-reperfusion led to RyR receptor injury, which promotes Ca^2+^ outflow in the sarcoplasmic reticulum. Together, these effects increase intracellular Ca^2+^ concentration rapidly. Reperfusion blood flow took away extracellular H^+^, and the concentration difference between intracellular and extracellular H^+^ further increased, enhancing the above effects. After reperfusion, the intracellular pH value gradually increased. Under the action of Ca^2+^, calpain was separated from its endogenous inhibitor, calpastatin. With the help of Ttm50, calpain migrated to the endoplasmic reticulum and was further activated in the high concentration of Ca^2+^ around the endoplasmic reticulum. NCX, Na^+^/Ca^2+^ exchanger; NHX, Na^+^/H^+^ exchanger; RyR, ryanodine receptors; LTCC, L-type Ca^2+^ channels.

Calpain is an intracellular non-lysosomal cysteine protease, one of the few proteases that the second messenger Ca^2+^ can directly activate. Due to the proteolysis ability of calpain being finite, calpain mainly plays a role in regulating rather than removing substrates in cells (15). Up to now, 15 types of calpain isoforms have been identified in the human body (The distribution of calpain in the human body is shown in Table 1) (15–17), among which calpain-1 (μ-calpain) and calpain-2 (m-calpain) are the most common, they are activated by micromolar and millimolar concentrations of Ca^2+^ *in vitro* experiments, respectively (18). Usually, intracellular Ca^2+^ concentration can only activate calpain-1 in physiological conditions. However, in pathological conditions, such as ischemia-reperfusion, intracellular Ca^2+^ concentration is higher than that in normal conditions. Abnormally elevated Ca^2+^ are combined with other mechanisms, such as phospholipids or phosphorylation, to activate calpain-2 (19). Calpain is usually in the form of an inactive enzyme that binds to calpastatin, an endogenous calpain inhibitor, to form an isomerase dimer, which can be located in the cytoplasm, endoplasmic reticulum, or mitochondria (20). Calpastatin inhibits calpain activation by binding to calpain through its calpain inhibitor domain (21). When the intracellular Ca^2+^ concentration was increased, calpain was separated from calpastatin and allowed to shift to the membrane (22). Calpain transfers to the cell membrane, binding to phospholipids to reduce the required Ca^2+^ concentration for activation or directly to the vicinity of Ca^2+^ channels. The activation of calpain is also affected by oxidative stress (23, 24). The reactive oxygen species produced by oxidative stress can directly inhibit ATPase, reduce the outflow of Ca^2+^ and destroy the calcium channels on the SR, resulting in the Ca^2+^ in the SR flowing into the cytoplasm, thereby activating calpain. After reaching the threshold of Ca^2+^ concentration for activation, the inactive enzyme was self-melted into the active form (18), stayed on the membrane, or was released into the cytoplasm to hydrolyze substrate proteins. Intracellular acidification significantly inhibits the activation of calpain (25). Therefore, calpain usually has the highest activity at the reperfusion stage after the pH value returns to normal. Calpastatin is another major factor affecting the activation of calpain in addition to the concentration of Ca^2+^ (26). Calpastatin plays a role in preventing calpain's excessive activation with the increased Ca^2+^ concentration. In contrast, calpastatin will be degraded by activated calpain or proteasome during myocardial ischemia-reperfusion (25, 27), issue in increased activation of calpain. Due to the activation of calpain being associated with Ca^2+^, which is an uneven distribution in cells, the subcellular localization of calpain may be related to its activation. Recent studies have found an interaction between the integrated membrane protein tiny tim50 (Ttm50) and calpain. This interaction promotes the directional movement of calpain to the Golgi body or endoplasmic reticulum, which is responsible for calcium storage. Moreover, Ttm50 increases the sensitivity of calpain to Ca^2+^ and enhances the activation of calpain (28). The possible mechanism of Ttm50 promoting calpain activation is: calpain is guided to move to a relatively high calcium environment in cells, and the conformation of calpain is changed to increase its affinity to Ca^2+^ so that calpain can be effectively activated in physiological conditions (19). Of course, there are other mechanisms to reduce the dependence of calpain activation on Ca^2+^ concentration, such as calpain phosphorylation and phospholipid-mediated calpain autolysis. Several phosphatidylinositols have been found to increase the autolysis of calpain on the cell membrane (29). ERK directly or indirectly activates calpain through phosphorylation of calpain or reduced Ca^2+^ concentration required for activation (30, 31). Up to now, the activation and regulation mechanism of calpain has not been fully understood, which needs further exploration (The activation mechanism of calpain during I/R is detailed in Figure 1).

**Table 1:** Distribution of calpain in the human body.

Gene	Chromosome	Expression preference
CAPN1	11q13	All cells
CAPN2	1q41-q42	Except erythrocytes
CAPN3	15q15.1-q21.1	Skeletal muscle
CAPN5	11q14	Most cells
CAPN6	Xq23	Embryonic muscles, placenta
CAPN7	3p24	Most cells
CAPN8	1q41	Gastrointestinal tracts
CAPN9	1q42.11-q42.3	Gastrointestinal tracts
CAPN10	2q37.3	Most cells
CAPN11	6p12	Testis
CAPN12	19q13.2	Hair follicles, skin
CAPN13	2p22-p21	Most cells
CAPN14	2p23.1-p21	Oesophagus
CAPN15	16p13.3	Most cells
CAPN16	6q24.3	Testis

## 3. Effect of calpain on myocardial ischemia-reperfusion injury

Calpain is a regulated protein that can perform limited protein hydrolysis to regulate the function of substrate (15). The Calpain system participates in maintaining the cell cycle, cell movement, and cell signal transduction and regulates cell differentiation, migration, and various programmed cell death processes. Calpain plays a crucial role in activation or degradation of proteins by signal-dependent cleavage of substrate proteins (32, 33). Calpain will be activated significantly during myocardial ischemia reperfusion (34). Calpain has inconsistent binding sites and cleavage sites on a wide range of substrates, which has a wide range of effects on cell biology. Therefore, basic and clinical researchers often include calpain in their research. Excessive activation or insufficient activation of calpain may lead to abnormal accumulation or excessive degradation of intracellular proteins, resulting in various cell damage and pathological conditions (19). It has been proved that myocardial I/R injury is related to the dysfunction of calpain (34–36). During I/R, a large amount of calpain is usually activated during the reperfusion period of normal intracellular pH value, at which the activity of calpain is the highest (25). The expression and activity of calpain in cardiomyocytes treated with hypoxia-reoxygenation and I/R were increased (34), and calpain activation on the cell membrane was positively correlated with myocardial injury (36). Inhibiting calpain activity by drug Inhibitors or genetic knockout reduces myocardial I/R injury (37, 38). The review of Hiroyuki Sorimachi details the research on the effect of intervening calpain on ischemia-reperfusion injury (15).

Cell death is a critical event in myocardial I/R injury. Since specific molecular pathways mediate PCD, there is the possibility of inhibiting these signaling pathways to reduce myocardial damage and dysfunction caused by I/R. It has been confirmed that apoptosis, mitochondrial-mediated necrosis, necroptosis, pyroptosis, parthanatos, and autophagy-dependent cell death play significant roles in myocardial I/R injury (5). The following contents discuss the function of calpain in various PCD (The effects of calpain on key molecules in various programmed cellular pathways during I/R are detailed in Table 2).

**Table 2:** The effects of calpain in programmed cell death.

The impacts of calpain in programmed cell death					
Programmed cell death	Molecule	The function of calpain	Acting site	Result	Reference
Apoptosis	Caspase-7	Activation	Cytoplasm	The large subunit of caspase-7 was cut into 17 and 18 kDa fragments, and the activity of caspase-7 was increased and induced apoptosis.	(39)
	Caspase-9	Devitalization	Cytoplasm	Cracking caspase-9 could not activate caspase-3 and blocked the activation of caspase-3 activated by dATP and cytochrome c.	(40)
	Caspase-3	Activation	Cytoplasm	Caspase-3 is activated by calpain-1 under oxidative stress.	(41)
	Caspase-12	Activation	Endoplasmic reticulum	Calpain split the N-terminal of caspase-12 to produce a 38 kDa fragment and lysed the C-terminal of caspase-3/7 to produce a 35 kDa fragment. Under the combined action of calpain and caspase-3/7, caspase-12 was significantly activated.	(42, 43)
	cIAP2、XIAP	Inhibition	Cytoplasm	Calpain reduced the expression of cIAP2 and XIAP, resulting in a significantly increased expression of caspase-3.	(44)
	BAX	Activation	Mitochondrial outer membrane	Calpain cleaves the N-terminal of BAX to produce an 18 kDa fragment. The cleavage of BAX promotes cytochrome C release and caspase-3 activation.	(45, 46)
	BID	Activation	Mitochondrial outer membrane	Calpain crack BID to generate tBID, which promotes the release of cytochrome C.	(47)
	BCL-XL	Devitalization	Mitochondria	Calpain cleavage BCL-XL produces a 25 kDa fragment that changes from anti-apoptosis to pro-apoptosis.	(43)
	P53	Devitalization	Nucleus	Calpain cleaves the N-terminal of P53 to produce a 46 kDa fragment, which reduces the stability of P53 and the effect of P53 in DNA damage-induced apoptosis.	(48)
Mitochondrial-mediated necrosis	Mitochondrial respiratory chain complex I	Devitalization	Mitochondrial matrix	Calpain cracks mitochondrial respiratory chain complex I decreased its activity by 60%, affecting mitochondrial function and metabolism.	(49, 50)
	ATP synthase subunit α (ATP5A1)	Devitalization	Mitochondrial matrix	Calpain directly binds with ATP5A1 and cuts it into multiple segments, resulting in decreased ATP synthase activity and increased ROS production in mitochondria.	(51, 52)
Autophagy	Beclin-1	Devitalization	Autophagosome precursor	Beclin-1 is cut by calpain to produce a 50 kDa fragment, which affects autophagy formation and reduces autophagy's occurrence.	(53)
	ATG-5	Devitalization	Nucleus	Calpain can cleave ATG-5 to reduce the formation of the ATG-5 and ATG-12 complex and reduce the formation of autophagosomes and autophagy.	(54)
	BIF-1	Devitalization	Golgi apparatus	Calpain cuts BIF-1 and promotes the migration of ATG9 and BIF-1 complexes on Golgi and some Golgi membranes into autophagosome precursors, promoting the formation of autophagosomes and the occurrence of autophagy.	(55)
Lysosome-dependent cell death	LAMP2	Degradation	Lysosome	Calpain promoted LAMP2 degradation and induced lysosome membrane permeabilization.	(56, 57)
	HSP70	Degradation	Lysosome	HSP70 stabilizes the lysosomal membrane by recycling damaged proteins. Calpain destroys this function and leads to lysosomal rupture or penetration.	(58)
Parthanatos	AIF	Activation	Mitochondria	Calpain cleaves AIF to activate it and opens the mitochondrial outer membrane channel by cleaving BAX to induce t-AIF to migrate to the nucleus.	(59)
	HSP70	Devitalization	Mitochondria	Hsp70 binds to AIF to reduce the nuclear translocation of AIF after stimulation. Calpain cleavage Hsp70 increases the nuclear translocation of AIF.	(60, 61)
Necroptosis	JNK-1	Activation	Cytoplasm	Calpain promotes JNK-1 activation, and the JNK-calpain complex induces RIPK-1 expression.	(62)
	STAT-3	Activation	Nucleus	Calpain increases RIPK-3 content by upregulating STAT-3 expression.	(63)
Pyroptosis	NLRP-3	activation	nucleus	Calpain upregulates NLRP-3 expression to form more inflammatory bodies with ASC and activates caspase-1 to induce pyroptosis.	(38)

### 3.1. Calpain and apoptosis

Apoptosis is a highly controlled process of cell death, independently carried out by entirely healthy or sublethally injured cells in response to physiological or pathological stimulation (64). Several experiments have shown that inhibition of apoptosis during I/R can reduce myocardial infarction size in mice (65–67). Apoptosis signal can be transmitted through the cell surface death receptor pathway (external pathway) and mitochondrial pathway (internal pathway). Caspase plays a vital role in apoptosis (68). The caspase family mainly has three subclasses: promoter (caspase-8, 9, 10), effector (caspase-3, 6, 7), and inflammatory factors (caspase-1, 4, 5). Calpain can directly act on caspase family proteins to influence apoptosis—for example, calpain cleavages caspase-3 and caspase-7 to activate apoptosis (39–41). Calpain also can cleavage caspase-12 to trigger apoptosis during endoplasmic reticulum stress (42). Caspase-3 also cleavages endogenous calpastatin after activation, enhancing calpain activity (69). Calpain can cleavage inhibitor apoptosis protein (IAP), such as cellular IAP2 (cIAP2), and X chromosome-linked IAP (XIAP), to increase the expression of caspase-3 and promote the occurrence of apoptosis (44). IAP acts as a caspase inhibitor in cells (70) and binds to caspase-3,7 and 9 through its baculovirus IAP repeat (BIR) domain to block caspase activation and negatively regulate intrinsic and extrinsic apoptosis pathways (71).

Calpain can affect apoptosis not only by directly interfering with caspases but also by cutting essential BCL-2 family proteins in the mitochondrial apoptotic pathway (45, 72, 73). BCL-2 family proteins are divided into three subtypes: pro-survival BCL-2 family members, such as BCL-2 and BCL-XL; the multi-BH-domain pro-apoptotic members, such as BAX, BAK, and BOK; the pro-apoptotic “BH3-only” proteins, such as BIM, BID, and BAD (74). The three subtypes interact to regulate the permeability of the mitochondrial outer membrane (75). The main process of mitochondrial apoptotic is BIM, BID activated by intracellular death signal, activated BIM, BID stimulates BAX, BAK and inactivates pro-survival BCL-2 protein. BAX or BAK can form a channel on the outer membrane of mitochondria. Cytochrome C, apoptotic peptidase activating factor 1 (APAF1), and deoxyribonucleotide complexes enter the cytoplasm through the channel and assemble into apoptotic body complexes (76), which activate caspase-9, and its downstream caspase-3 induces apoptosis (77). Under the stimulation of ischemia-reperfusion injury, calpain-1 cuts BID into t-BID to activate it (47). T-BID can induce apoptosis by affecting mitochondrial outer membrane permeability in the following three ways: (i) Mediated BAX and BAK oligomerization; (ii) Self-forming homodimer; (iii) Induced mitochondrial permeability transition pore (mPTP) opening and remolded mitochondrial inner membrane (78). Decreasing calpain-1 expression by specific siRNA can significantly decrease isoproterenol-induced expression of Bax, t-Bid and cytochrome C release in cardiomyocytes (79). In addition, calpain can also cleave the N-terminal of BAX into an 18 kDa fragment to activate BAX and promote cytochrome C release from mitochondria to induce apoptosis (45).

**Figure 2:**
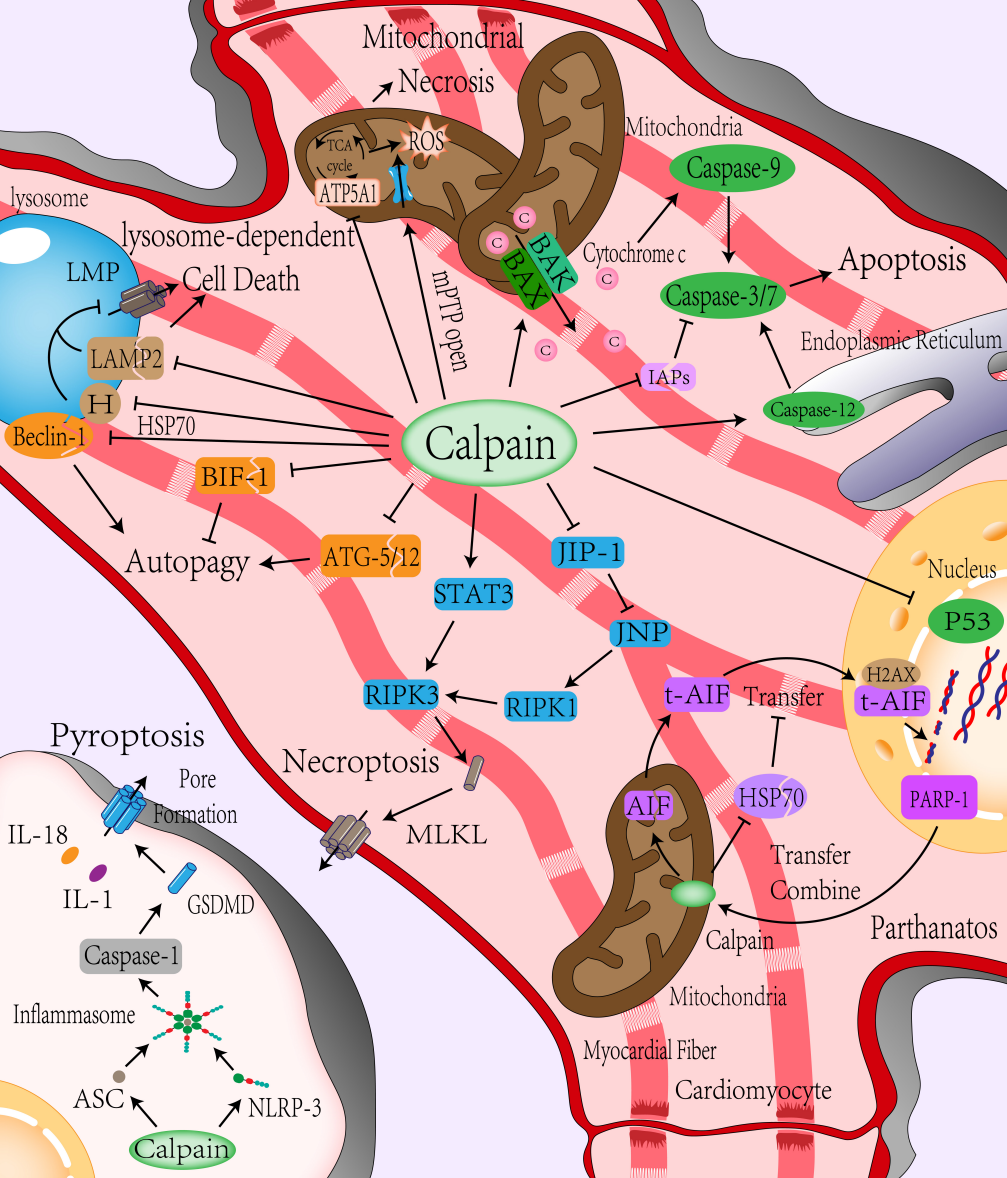
Role of calpain in programmed cell death. (i) Apoptosis: Calpain can directly lyse and activate caspase-3,7, and 12 to mediate cell apoptosis and reduce IAPs’ inhibition of caspase activity by cracking IAPs. Calpain can directly activate BAX/BAK or indirectly activate BAX/BAK by activating BID to induce the release of proapoptotic substances such as cytochrome C in the formation channel of the mitochondrial outer membrane to mediate the occurrence of apoptosis and reduce the response of cells to DNA damage by inhibiting P53. (ii) Mitochondrial-mediated cell necrosis: Calpain can directly promote mitochondrial inner membrane mPTP channel opening, cleavage of ATP synthase, result in energy disorders, ATP synthesis decreased, increased ROS generation, aggravate intracellular energy conflicts, and promote mitochondrial-mediated cell necrosis. (iii) Autophagy: Calpain inhibits autophagy by cleavage of Beclin-1 and autophagy-related protein ATG-5/12, which is involved in autophagosome formation, or inhibits apoptosis by cleavage of apoptosis-related protein BIF-1 to promote autophagy reversely. (iv) Lysosome-dependent cell death: Calpain can affect the stability of the lysosomal membrane by cleavage of LAMP2 and HSP70, causing lysosomes to permeate or rupture and mediating lysosome-dependent cell death. (v) Parthanatos: DNA damage during ischemia-reperfusion leads to PARP-1 activation, which is transferred to the mitochondrial membrane to activate AIF by Calpain cleavage. AIF is activated and then transferred from mitochondria to the nucleus through the cytoplasm. DNA is decomposed into large segments by calpain with the help of histone H2AX and cyclophilin. Calpain also cleaves HSP70 to reduce the stability of AIF and helps AIF transfer to the nucleus. (vi) Necroptosis: Calpain can increase the expression of RIPK1 by cleaving JIP-1, inhibiting JNP, and promoting the expression of RIPK3 by increasing the expression of STAT3, aggregation of MLKL leading to necroptosis. (vii) Pyroptosis: Calpain can up-regulate the expression of NLRP-3 to form more inflammatory bodies with ASC, then activate caspase-1 to mediate pyroptosis. mPTP, mitochondrial permeability transition pore; ATP5A1, ATP synthase subunit α; IAPs, inhibitor apoptosis protein; ATG, autophagy-related proteins; BIF-1, Bax-interacting factor 1; LMP, lysosomal membrane permeabilization; HSP70, Heat-shock protein70; LAMP2, lysosomal associated membrane protein 2; PARP-1, poly ADP-ribose polymerase 1; AIF, apoptosis-inducing factor mitochondria-associated 1, also known as AIFM1; H2AX, histone; RIPK1/3, receptor-interacting protein kinases 1/3; MLKL, mixed line kinase domain like; JNK1, c-Jun-N-terminal kinase 1; JIP-1, JNK-interacting protein-1; STAT3, signal transducer and activator of transcription 3; GSDMD, gasdermin D; NLRP-3, nucleotide-binding oligomerization domain-like receptor with a pyrin domain 3; ASC, apoptosis-associated speck-like protein.

However, some researchers have shown that calpain-2 can be used as a negative regulator of caspase activation and apoptosis. Activation of calpain-2 not only failed to cleave and activate caspase-3 but also inhibited it, which was mainly related to the inactivation of caspase-9 by calpain cleaving, blocking the activation of caspase-3 by dATP and cytochrome C (40). In addition, calpain can hydrolyze P53 protein to inhibit apoptosis induced by DNA damage (48). The content of P53 protein increased rapidly during myocardial I/R. Inhibition of P53 significantly attenuated the I/R injury (80). In summary, there may be two effects of calpain on apoptosis induced by myocardial I/R, and different calpain subtypes may have different effects on caspase (The mechanism of calpain affecting apoptosis is shown in Figure 2).

### 3.2. Calpain and mitochondrial-mediated necrosis

Mitochondrial-mediated necrosis is caused by intracellular microenvironment disorders, such as severe oxidative stress and intracellular Ca^2+^ overload, usually presented as necrosis (81). The critical event of mitochondrial-mediated necrosis is the opening of mitochondrial permeability transition pore (mPTP) that is located on the mitochondrial inner membrane. mPTP opening is mainly mediated by Ca^2+^ concentration and mitochondrial membrane potential. An increase in Ca^2+^ concentration and depolarization of mitochondrial membrane potential will promote mPTP opening. The mPTP opening leads to increased permeability of the mitochondrial inner membrane that results in a dissipation of the proton gradient across the inner membrane. Water entered the mitochondrial matrix along the permeability gradient, resulting in mitochondrial swelling. Since the area of the mitochondrial inner membrane is much larger than that of the outer membrane, the expansion of the inner membrane is often accompanied by the loss of the integrity of the outer membrane. BAK and BAX mediate the formation of large enough pores in the outer membrane to allow the extrusion of the inner membrane. Moreover, proton leakage leads to the disappearance of mitochondrial membrane potential and the inhibition of ATP synthase leads to oxidative phosphorylation uncoupling, which lead to mitochondrial dysfunction. Dysfunctional mitochondria cannot produce enough energy to close mPTP, eventually leading to necrosis (82). The specific mechanism of necrosis caused by the opening of mPTP is not precise, which may be due to the increase of glycolysis rate cannot offset the depletion of cell energy and leads to the acidification of the cytoplasm. ATP deficiency damages the enzymes that pump Na^+^, H^+^, and Ca^2+^ out of the cell and the enzymes that pump Ca^2+^ into the endoplasmic reticulum. The sharp loss of Na^+^ and Ca^2+^ gradient inside and outside the cell causes cell membrane collapse and cell death with necrosis characteristics (82). Compared with apoptosis, the process of intracellular energy consumption (such as DNA repair and protein translation) during necrosis does not terminate. On the contrary, energy consumption has been intensified, and the intracellular energy contradiction has been aggravated. Therefore, a sharp decrease in ATP is also a sign of necrosis (5). Calpain exists not only in the cytoplasm but also in the mitochondria. Studies have shown that calpain activity in cardiac mitochondria increases during I/R (83). Ca^2+^ and ROS are considered to be two key activators for mPTP opening (84). Calpain induces Ca^2+^-induced mPTP opening through cleavage of complex I subunits (ND6 and NDUFC2) on the electron transport chain (85), The destruction of complex 1 leads to sensitization of mPTP (86). In addition, the increased calpain activity in mitochondrial can impair mitochondrial respiratory chain complex I (49, 50) and ATP synthase, such as ATP synthase subunit α (ATP5A1), these will cause mitochondrial dysfunction and increase the production of reactive oxygen species (ROS) (51, 52). Recent experiments have proven that overexpression of calpain inhibitors can maintain the activities of ATP5A1 and ATP synthase, prevent mitochondrial ROS production and reduce necrosis after hypoxia and reoxygenation (34). In summary, calpain not only promotes the opening of mPTP but also acts on ATP synthase in the process of mitochondrial-mediated necrosis, affecting mitochondrial energy production, aggravating intracellular energy contradiction, and promoting cell necrosis (The mechanism of calpain involving mitochondrial-mediated necrosis is shown in Figure 2).

### 3.3. Calpain and autophagy-dependent cell death

Autophagy-dependent cell death is a PCD that depends on autophagy and its components (81). Autophagy, a function of conservative evolution, transports endogenous or exogenous substances in the cytoplasm to lysosomes for degradation. It plays a crucial role in quality control, stress alleviation, and recovery of cell homeostasis. It is usually used as a means of survival (87). However, in some pathophysiological environments, autophagy also can cause cell death, known as autophagy-dependent cell death (88). It is unclear whether autophagy-dependent cell death is caused by the high autophagy rate (excessive catabolism of intracellular components) or the change in the nature of the autophagy process itself. Therefore, this paper mainly introduces the role of calpain in autophagy-related molecules. Autophagy is mainly divided into macroautophagy, microautophagy, and chaperone-mediated autophagy. Autophagy usually refers to macroautophagy (88). Autophagy occurs mainly through two steps: autophagosome formation and transfer of autophagic substrates to lysosome (89). Beclin-1, part of the class III phosphatidylinositol-3-kinase (PI3K) complex, is responsible for phosphatidylinositol (PI) phosphorylation to form autophagosome precursors, which is indispensable in the formation of autophagosomes (90). Autophagy during reperfusion is closely related to Beclin-1. Autophagy is attenuated in Beclin-1 knockout mice during myocardial reperfusion (91). Calpain also plays a particular role in autophagy (92). Calpain can lyse Beclin-1 to reduce autophagy activity (53). Calpain also cleaves autophagy-related proteins (ATGs) to influence autophagy (54), ATGs are closely related to autophagosome maturation and autophagy (93), and ATGs play different roles in different stages of autophagy. For example, ATG-5 is involved in forming autophagosomes that encapsulate proteins or organelles. ATG-5 is the cutting substrate of calpain. After being cut, ATG-5 not only inhibits the formation of autophagosomes but also translocates to mitochondria and binds with BCL-XL, resulting in the release of cytochrome C (94). In addition, calpain affects autophagy by cleaving apoptosis-related molecules such as BAX-interacting factor 1 (BIF-1) (55). Although most studies have shown that calpain affects autophagy, most experiments only focus on the effect of calpain on the autophagy mechanism. Autophagy and autophagy-dependent cell death and the relationship between calpain and autophagy-dependent cell death need further research (The mechanism of calpain affecting autophagy-dependent cell death is shown in Figure 2).

### 3.4. Calpain and lysosome-dependent cell death

Lysosome-dependent cell death is a PCD caused by the permeability of the lysosome membrane; proteolytic enzymes are released. The upstream mechanism of lysosomal membrane permeabilization (LMP) has not been fully understood. Previous studies have indicated that LMP mainly occurs after mitochondrial outer membrane permeabilization, which constitutes an intrinsic apoptosis-associated phenomenon. However, under certain experimental conditions, lysosomal membrane permeabilization can precede mitochondrial permeability, thereby mediating lysosomal-dependent cell death. The mechanism may be related to the recruitment of BAX to the lysosome membrane for pore formation (81). The stability of the lysosome membrane is mainly associated with the cholesterol content of the lysosome and member 1A of heat shock protein family A (HSPA1A; best known as HSP70) (95). Calpain affects lysosomal membrane permeability through various mechanisms (96). Calpain can cleave lysosomal associated membrane protein 2 (LAMP2) on the lysosomal membrane. High glycosylation of LAMP2 can prevent the lysosomal membrane from being dissolved by lysosomal enzyme (97). When LAMP2 is cleaved by calpain, lysosomal membrane permeabilization and proteolytic enzyme release lead to lysosome-dependent cell death (56, 57). Heat shock protein 70 (HSP70) plays a crucial role in maintaining lysosome stability (98). It can increase the activity of acid sphingomyelinase (ASM) by binding to the lysosomal anionic phospholipid bis monoacylglycerol phosphate (BMP) and promote the conversion of sphingomyelin from lysosome membrane to ceramide. Ceramide is one of the sphingolipids, these lipids serve structural roles in biomembranes (99). On the lysosomal membrane, ceramide enhances membrane acyl chain order and increases lateral packing of lipids *in vitro* (100). The content of ceramide affects the spatial conformation of lysosomal membrane, increases the fusion ability of lysosomal membrane with intracellular vesicles and cell membrane, and thus affects the composition and volume of lysosomal membrane, which is helpful to maintain the stability of lysosome (101). Interestingly, HSP70 is a calpain substrate *in vivo* and vitro (98, 102). HSP70 is more likely to be cleaved by calpain after carbonylation. Hsp70 is the regulator of calpain-mediated lysosomal permeabilization or rupture after I/R injury (58). Although there is no direct evidence to prove that lysosome-dependent cell death mediates the cell death of cardiomyocytes during ischemia-reperfusion injury, it may be hidden in autophagy-dependent cell death or apoptosis due to its close relationship with autophagy. Therefore, it is still described here (The mechanism of calpain affecting lysosome-dependent cell death is shown in Figure 2).

### 3.5. Calpain and parthanatos

Parthanatos is a PCD mediated by poly ADP-ribose polymerase 1 (PARP-1) and is mainly activated by oxidative stress-induced DNA damage and chromatin decomposition (103). PARP-1 is a chromatin-related nuclear protein that plays a vital role in the repair of DNA single-strand or double-strand breaks. PARP-1 promotes DNA repair by identifying DNA ends and uses nicotinamide adenine dinucleotide (NAD^+^) and ATP to form poly (ADP-ribose)-sylation to change chromatin structure. However, DNA base modification affected by oxidative stress, hypoxia, and inflammatory stimuli can lead to excessive activation of PARP-1 (104). Over-activated PARP-1 consumes much NAD^+^ and ATP, leading to mitochondrial transmembrane potential dissipation. Over-consumption of intracellular energy leads to energy collapse and amplification of necrosis (105). Some scholars believe that parthanatos is a step before cell death. It is unclear whether specific inhibition of parthanatos can benefit myocardial I/R injury. Apoptosis-inducing factor mitochondria-associated 1 (AIFM1, also known as AIF) is necessary for parthanatos to perform. Over-activated PARP1 binds to AIFM1, leading to the transfer of AIFM1 from mitochondria to the nucleus and causing lethal chromatin dissolution (106). Calpain is directly involved in the occurrence of parthanatos in cells. PARP-1 is activated after DNA damage and is transferred to the mitochondrial membrane after activation. Calpain-1 in the mitochondrial intermembrane space is used to shear and activate AIF (107). The activated AIF is transferred to the nucleus, decomposed DNA into large fragments with histone H2AX and acyclic proteins (59). The release of t-AIF from mitochondria also requires calpain cleavage of BID to activate BAX formation channels (108). In addition, HSP70 can increase the stability of AIF and reduce nuclear transfer (60). Calpain can promote the transport of AIF from mitochondria to cytoplasm or nucleus by degrading HSP70 (61). Therefore, calpain may be synergistic in the induction of parthanatos during ischemia-reperfusion injury (109) (The mechanism of calpain affecting parthanatos is shown in Figure 2).

### 3.6. Calpain and necroptosis

In the past, necrosis was believed to be an accidental or passive type of cell death. However, with the deepening of research, it was found that cell death factors such as tumor necrosis factor-α (TNF-α) could cause a kind of “programmed” necrosis, namely necroptosis (110). After the binding and activating of cell death factors with corresponding receptors, receptor-interacting protein kinase 1 (RIPK1) and receptor-interacting protein kinase 3 (RIPK3) were recruited into the cells to form a “necrosome.” Subsequently, the mixed line kinase domain like (MLKL) was activated (5) and shifted to the cell membrane to create a channel leading to membrane permeabilization (111). Studies have shown that calpain regulates necroptosis (62). Calpain can degrade c-Jun-N-terminal kinase 1 (JNK1) specific inhibitor, JNK-interacting protein-1 (JIP-1), induce JNK activation, and increase the expression of RIPK1 (62). Calpain also can increase the expression of RIPK3 by affecting the phosphorylation of signal transducer and activator of transcription 3 (STAT3), promote the phosphorylation of MLKL, and induce cardiomyocyte necroptosis (63). Inhibition of calpain can reduce the number of activated AIF and the occurrence of necroptosis (112) (The mechanism of calpain affecting necroptosis is shown in Figure 2).

### 3.7. Calpain and pyroptosis

Pyroptosis is a form of PCD closely related to innate immune response, characterized by cell membrane permeabilization and extracellular release of inflammatory factors. The central part of Pyroptosis is the activation of gasdermin D (GSDMD). GSDMD has an inhibitory -COOH terminal and death-promoting -NH_2_ terminal, which interacts to maintain the inactivation of GSDMD (113). Cells form inflammatory bodies after identifying various stressors (such as DAMPs and PAMPs), which recruit and activate inflammatory caspases (108). Inflammatory caspases can cut and activate GSDMD. After GSDMD activation, pores are formed on the cell membrane, releasing cellular contents, such as inflammatory factors, and ultimately Pyroptosis. Research has shown that inhibition of GSDMD activation during myocardial ischemia-reperfusion can reduce the occurrence of Pyroptosis and myocardial injury (114). The occurrence of Pyroptosis requires the involvement of inflammatory caspases such as caspase-1 and inflammatory bodies (115, 116). In myocardial I/R, calpain can up-regulate the expression of NLRP-3, making it form more inflammatory bodies with ASC to activate caspase-1 and induce Pyroptosis, causing myocardial injury (38) (The mechanism of calpain affecting Pyroptosis is shown in Figure 2).

## 4. Effect of calpain on cardiac remodeling after myocardial ischemia-reperfusion injury

During reperfusion, intracellular Ca^2+^ overload and increased pH increased calpain activity, leading to PCD by hydrolyzing various proteins. This is a feature of acute reperfusion injury (6). Calpain is at the regulatory point of positive feedback in most death processes. The increase of Ca^2+^ caused by I/R activates calpain, resulting in cell damage. The damage promotes further activation of calpain, which in turn aggravates the injury and eventually leads to PCD. Therefore, inhibiting calpain may reduce the heart damage caused by I/R, which has been verified in many experiments using drugs or gene inhibitors to reduce the activity of calpain (37, 38).

Calpain not only plays a significant role in acute myocardial I/R injury but also plays a vital role in myocardial remodeling after injury. Although the mechanism of over-expression of calpain remains to be determined in the late reperfusion period, it is reported that calpain remains high in the late reperfusion period (117, 118). High calpain expression in late reperfusion is involved in adverse remodeling after myocardial infarction. Inhibition of calpain within 24 h after transient coronary occlusion produces cardioprotective effects independent of the acute injury, reduces myocardial hypertrophy and fibrosis induced by I/R and attenuates adverse cardiac remodeling after infarction (119). Overexpression of calpastatin inhibits angiotensin-induced myocardial and perivascular inflammation and fibrosis (120). Removal of calpain-1 and calpain-2 by specific knockout of calpain small subunit 1 (Capn4) in cardiomyocytes reduces ventricular dilatation, dysfunction, and mortality after 30 days of infarction (121). Similar results were obtained in transgenic mice overexpressing endogenous calpastatin (117). On the contrary, deleting calpastatin increases calpain-induced cardiac remodeling, systolic dysfunction, and mortality after infarction (122). It can be seen that calpain harms myocardial remodeling. In general, inhibition of calpain can reduce myocardial damage caused by ischemia-reperfusion.

However, there are also reports that calpain inhibition can adversely affect cardiomyocytes (26, 123). In a recent study, overexpression of calpastatin resulted in impaired scar healing, cardiac rupture, and increased mortality (124). In addition, cardiac-specific knockout of the calpain subunit Capn4 affects isoproterenol and hemodynamic pressure-induced cardiac remodeling in mice, attenuating ventricular function (123).

## 5. Discussion

Because calpain the mechanism of action has not been identified and the basic experimental results of calpain intervention are unstable, the treatment of calpain intervention has not been widely used in the clinic. There may be the following reasons for the different intervention results in calpain in basic experiments: (i) At present. There is still a lack of specific inhibitors of calpain. Most studies have adopted a wide range of non-selective calpain inhibition methods, ignoring the different subtypes of calpain that may have different effects. Javier Inserte et al. also put forward similar views (6); (ii) Calpain regulates many essential proteins of the heart, and the impact of calpain on these proteins is often ignored in the experiment. Among them, the most famous is spectrin. Spectrin is a critical membrane cytoskeletal maintenance protein, which plays a crucial role in assembling actin cytoskeleton, cross-linked actin filaments in various cell types, and scaffolds for assembling large protein complexes involving structural integrity, mechanical sensation, and cell signal transduction (125). The calpain-dependent protein hydrolysis of spectrin is closely related to cell death (15); (iii) Some death mechanisms have not been fully clarified. On the one hand, the existing death mechanisms are not sufficiently detailed—for example, the relationship between autophagy and autophagy-dependent cell death. Calpain can inhibit the occurrence of autophagy by degrading autophagy-related proteins, but in some cases, the damage after inhibition of calpain is more robust than before. The possible reason is that calpain leads to increased autophagy or changes in the nature of autophagy, resulting in autophagy-dependent cell death or lysosome-dependent cell death; On the one hand, new ways of death are still being discovered, such as copper death, cell death caused by acylation of TCA cycle proteins (126), some key proteins in TCA cycle have also been shown to be substrates of calpain in some studies (49, 50), calpain may also play a role in the occurrence of copper death; (iv) Disorder of substrate cleavage by calpain. Calpain cleavage does not target a specific molecule, which is a complete result after affecting all molecules. For example, calpain eventually leads to apoptosis, which may directly affect apoptosis-related proteins to mediate apoptosis or inhibit autophagy-related proteins from promoting apoptosis. The results may be similar. Cells eventually undergo apoptosis. However, the intermediate pathways may be different. We cannot quantify whether this apoptosis is mediated by calpain activating apoptosis-related proteins or inhibiting autophagy-related proteins; both work together or are mediated by other pathways. Therefore, if the ample cleavage of calpain is ignored, the isolated environment only considers the influence on one or more of the pathways, which may lead to a significant deviation in the experimental results.

Studies have reported that the calpain cleavage site is structurally dependent, not sequence-dependent. Calpain cleavage is uncertain. Why calpain eventually mediates apoptosis rather than autophagy or pyroptosis in some cases? Is there a pathway affecting the cleavage trend of calpain? However, there is no report on the related mechanism. If it can be found that something affects the tendency of calpain cleavage, this may have a significant impact on the future treatment of calpain. In addition, since we cannot ignore the extensive cleavage caused by calpain, can we analyze the action environment of calpain? With the development of proteomics, spatial transcriptomics, and protein structure prediction, we may know all proteins that can be cut by calpain at a particular time. Suppose apoptosis-related proteins account for the vast majority of the proteins that can be cut by calpain in cells at a specific time. Will we inhibit apoptosis by inhibiting calpain at this time? Therefore, we guess that the action time window of calpain may also significantly impact the experimental results. There is still a large blank in the study of calpain. With the progress of science and technology and the further understanding of various death mechanisms, the regulatory role of calpain will be further analyzed. The mode of action, targeted therapy, and treatment time of calpain may be essential research directions in the future.
